# Parents’ information needs, self-efficacy and influences on consulting for childhood respiratory tract infections: a qualitative study

**DOI:** 10.1186/1471-2296-14-106

**Published:** 2013-07-28

**Authors:** Jenny Ingram, Christie Cabral, Alastair D Hay, Patricia J Lucas, Jeremy Horwood

**Affiliations:** 1 School of Social and Community Medicine, University of Bristol, Bristol BS8 2BN, UK; 2 School of Social and Community Medicine, University of Bristol, Bristol BS8 2PS, UK; 3 School for Policy Studies, University of Bristol, Bristol,BS8 1TZ, UK

**Keywords:** RTI, Childhood cough, Qualitative, Health belief model, Self-efficacy

## Abstract

**Background:**

Acute respiratory tract infection (RTI) is the most common reason why parents consult primary care in the UK. Little is known about parents’ perceptions of what may help them to make an appropriate decision to consult when their child is ill and how to improve self-care.

Using qualitative methods, this study aimed to explore parents’ views on support and information needs prior to consulting when children have RTIs with cough, and identify the triggers and barriers to consulting primary care.

**Methods:**

7 focus groups and 30 semi-structured interviews were held with 60 parents (with children aged 5 months - 17 years) from a range of socio-economic backgrounds. Topics discussed were informed by the Health Belief Model, and explored parents’ concerns and beliefs about susceptibility and severity of RTIs, beliefs about the triggers and barriers to consulting, and information and support seeking behaviour undertaken before consulting primary care. Discussions were audio-recorded, transcribed and analysed using thematic methods.

**Results:**

Parents from all socio-economic backgrounds sought information from a wide range of sources about RTIs in children in order to identify which of their child’s symptoms should be of concern and trigger a visit to the doctor. The perception of threat to a child of RTI (with cough) was increased with more severe illness and by perceived susceptibility to illness of a particular child; whilst experience with other children increased parental efficacy to cope with childhood cough at home. Psychological models of health behaviour informed the understanding of cultural beliefs and attitudes that underpin health related behaviours.

**Conclusion:**

A wide range of perceptions influence the likelihood that parents will seek help from primary care for a child with cough; these perceptions are similar across socio-economic groups. Parents’ experience, confidence and efficacy influence the likelihood of consulting primary care for their child’s RTI. Parents would value consistent advice from a trusted source that addresses common concerns and supports home care and decision making about help seeking.

## Background

Childhood acute respiratory tract infections (RTIs) are the most common reason for parents to consult primary care in the UK [1]. RTIs in children are problematic, as despite being largely self-limiting illnesses, they cause significant disruption to children’s health and family life, and are occasionally associated with severe complications such as pneumonia and empyema. They are therefore a significant cause of anxiety for parents [2,3], primary health care resource utilisation, and antibiotic use. The overall annual National Health Service (NHS) cost burden of childhood RTIs is not known, but the primary care costs for acute cough-associated RTI has been estimated at £31m, with most costs arising from primary care consultations [4].

Thus, parental decision making to consult has significant implications for primary care workload. Challenges for parents include deciding whether, and at what point, they should consult their general practitioner (GP). Attendance at the GP practice is dependent on parental subjective interpretations of the perceived severity of their child’s symptoms [5] and their perceptions of the benefits of consulting [6]. Parents’ assessment of illness severity may be influenced by their perceived threat of an illness and their concerns regarding their responsibility to ensure their child’s safety [2,6]. These help seeking behaviour dilemmas can be explained using psychological models of health behaviour. One of these, the Health Belief Model, is based on the premise that an individual’s belief in a personal threat (susceptibility to and severity of illness), together with beliefs about the benefits and costs of a health action, will predict the likelihood of that behaviour [7,8]. Perceptions of benefit relate closely to perceived efficacy, both in the health action (treatment efficacy) and in themselves to carry out this action (self-efficacy) [7,9]. This concept has been used widely in health behaviour studies to explain the complex mix of psychological and social factors that influence consulting behaviour [10,11] and parental decisions to use health care for their children in particular [12].

A systematic review of the support needs of parents making child health decisions found that parents use a variety of sources and their trust in the information was dependent on the perceived credibility of the source [13]. Two qualitative focus group studies reported that parents sought help from families and friends following negative experiences of seeking advice from health professionals [14], and found that younger mothers sought information on-line to support their own beliefs and to counter conflicting advice offered by health professionals [15]. Sociological reports suggest that the public may become more sceptical of medical authority due to increased access to medical knowledge via the internet [16].

Little is still known about parents’ perceptions of when they need to consult, where to get information about self-care and the triggers and barriers to consulting primary care. This study (part of the “TARGET” NIHR Programme for Applied Research) aimed to explore parents’ views on support and information needs prior to consulting when children have RTIs to inform an intervention to support future parental decision-making.

## Methods

### Participants and methods

Between October 2010 and May 2011, seven focus groups and 30 interviews were conducted. The focus groups aimed to investigate parents’ pre-consultation beliefs and behaviours and these parents were recruited from the community. The interviews aimed to investigate parents’ views and experiences following recent consultations for children with cough recruited through primary care practices. The focus groups each comprised 4-6 mothers and were recruited from pre-existing groups, including parent and toddlers, ante-natal class friendships, and groups associated with schools and community organisations. Parents with children between 3 months and 12 years were invited to take part. Those who only had children less than 3 months of age were excluded because they can have different clinical features (e.g. less likely to develop fever) and are at greater risk for serious complications than older children [17]. Parents with older children were excluded because they are less likely to consult [17] and older children may play an increasing role in decision making, whereas this study was concerned with parental decision making. Purposive sampling was used to select participants to capture maximum variation in views and experiences. The groups were stratified in relation to parents’ socio-economic situation (SES) and age of children, and only mothers volunteered for the focus group discussions.

Eligible parents for the interviews were identified through a search of patient records, in six GP practices, for those who had consulted in the previous 3 months for a child with a respiratory infection; where more than 60 eligible parents were identified by a practice, 30 letters of invitation were sent to the parents of children who had consulted the doctor (for any cause) most frequently during the past 12 months and 30 letters were sent to parents of children who had consulted least frequently during the same period in order to include parents with a range of consulting frequencies. Twenty-one mothers and two fathers volunteered and were interviewed. In order to increase the socio-economic diversity of parents in the interview sample, 7 younger mothers who had participated in the focus groups were also recruited for interviews.

### Procedure

Focus groups were conducted in a range of non-clinical community settings. Most interviews took place at patient’s homes with a few in other non-clinical settings. All participants received both written and verbal information about the research and provided written informed consent before the group or interview. The focus groups were facilitated by three researchers, one led the discussion using open-ended questioning techniques to elicit participants’ own experiences and views, and ensure all participants had an opportunity to take part; one summarised the discussion on a flip chart to allow for group checking and reflection during the session; and the third audio-recorded the session and noted down who was speaking to aid transcription. Each focus group lasted between 60-85 minutes. Interviews were conducted by one researcher and lasted between 30 and 90 minutes. There were separate topic guides for the focus groups and for the interviews. The topic guides for the focus group investigated parents’ pre-consultation beliefs and behaviours. Topic guides for the interviews investigated parents’ perceptions and experiences of primary care consultations when their child had a cough. Topic guides, informed by the Health Belief Model, were used to facilitate the focus group discussions and semi-structured interviews (See Table 1).

**Table 1:** Outline topic guides

**Focus groups**	**Interviews**
• Pre-consultation information and	• Trigger to consultation
• support seeking	• Beliefs about susceptibility and severity of RTI
• Triggers and barriers to consulting	• Beliefs about benefits of consulting
• Beliefs about severity of RTIs	• Information sought during consultation
• Information needs	• Parent self-efficacy in caring for their child post consultation

### Analysis

Focus group data collection and analysis were conducted in parallel and groups continued until data saturation was reached and no new themes were arising from the data [18]. Preliminary findings and questions from early focus groups were raised at later groups for further discussion. Findings from the focus groups helped to influence the topic guide for the interviews which also continued until data saturation was reached. Focus groups and interviews were audio-recorded, fully transcribed, anonymised, checked for accuracy and then imported into the software package NVivo8. Thematic analysis [19] , using the constant comparison technique [20], was used to scrutinise the data to identify and analyse patterns across the dataset. Transcripts were examined on a line-by-line basis with codes being assigned to segments of the data to provide insight into the participants’ views and beliefs and an initial coding frame developed. The process of constant comparison allowed for the coding frame to be modified as analysis developed, and allowed us to generate new themes, re-classify existing themes and incorporate them within others. Two focus groups and two interviews were coded independently by two researchers; any discrepancies were discussed within the research team to achieve a coding consensus and maximise rigour. The index of multiple deprivation (IMD) score for the parents’ home post-codes was obtained and they were categorised into IMD quintiles. These quintiles were then used to group parents into those living in high-SES (affluent) areas (two least deprived IMD quintiles), mid-SES areas (middle IMD quintile) and low-SES (deprived) areas (two most deprived IMD quintiles) and a comparison of themes by SES was carried out.

## Results

### Sample description

In total 60 parents took part in the study; 30 in the focus groups only, 23 in interviews only and 7 in both. Table 2 describes the wide range of socio-economic areas, education level and age of the parents who took part. Most families had 1 or 2 children ranging in age from 5 months to 17 years old; most parents were of white-British ethnicity, 2 parents were white-other ethnicities and 5 were of non-white ethnicities (3 Black, 2 Asian, 1 mixed).

**Table 2:** Characteristics of mothers involved in the focus groups and interviews

		**Focus groups only (n = 30)**	**Focus group and interview (n = 7)**	**Interviews only (n = 23)**	**Total (n = 60)**
**Socio-Economic Status (SES) – measured as Index of Multiple Deprivation (IMD) quintile from home post code**	1 (most deprived)	7	6	3	16
	2	5	0	6	11
	3	10	0	3	13
	4	2	0	4	6
	5 (most affluent)	6	1	7	14
**Education**	Left school <16yrs	1	3	0	4
	Schooling to 16 yrs	7	3	7	17
	Schooling to 18 yrs	9	0	3	12
	Graduate degree	1	0	9	10
	Post graduate	12	1	4	17
	degree				
**Age of parent**	<25	4	4	0	8
	25-34	12	3	7	22
	35-44	7	0	12	19
	45+	7	0	4	11
**Number of children**	1	12	3	10	25
	2	15	4	10	29
	3+	3	0	3	6
**Age of youngest child**	<2yrs	10	3	8	21
	2-4yrs	6	3	7	16
	>4yrs	14	1	8	23
**Frequency of consultations with GP (for youngest child) [self-reported]**	1-3 per yr	19	0	9	28
	4-6 per yr	6	2	7	15
	7-12 per yr	4	2	3	9
	>12 per yr	1	3	4	8

**Figure 1:**
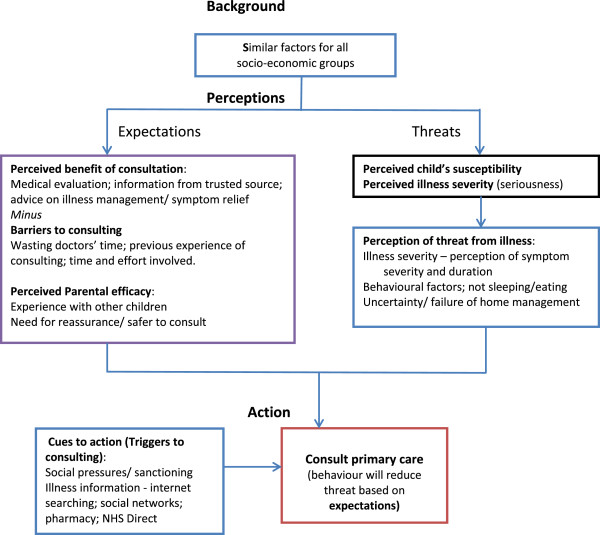
HEALTH BELIEF MODEL: How theme summaries influenced the likelihood that parents consulted primary care for childhood cough.

Themes developed from the analysis related to the Health Belief Model were: perceptions of personal threat from illness; the perceived benefits of, triggers and barriers to consulting for a cough; and perceived parental efficacy as shown in Figure 1. Quotes from parents illustrate the points being made and they are identified by high, mid or low socio-economic status (SES) and ages of their children.

### Perceptions of threat from illness

#### Perceived illness severity

The perceived threat of a cough to a child included a combination of the severity of the illness and the susceptibility of a particular child to developing a cough. Parents from all backgrounds did not feel confident about caring for a child at home with a persistent cough. They suggested that a cough alone was less serious, but in combination with fever, croup, breathing difficulty or a child not eating, it was felt to be more serious and likely to cause them to seek help from primary care. Their perception of the risk of the illness was linked to the child’s symptoms and their own experience.

*“I’m more worried about a high temperature more than anything”.* (mid-SES, 2 children 9 & 12y)

*“I think a cough is about a couple of weeks normally. And if it’s prolonged then I go to the doctor.*” (high-SES, 2 children 11 & 13y)

*“Also when they’re just not themselves. So some kids (…) are sort of quite lifeless and, you know, exhausted or sleepy or whatever, then they’re obviously feeling ill”.* (high-SES, 2 children 11 & 13y)

*“(…) kind of the ferocity of the cough, (..) is it kind of – is it making them really kind of go red in the face when they are coughing?* “ (mid-SES, 1 child 11m)

*“ He’ll eat anything, so if he’s off his food I know it’s something serious”.* (mid-SES, 2 children 1 & 7y)

*“More of a harsh cough as well like.(…) like a smoker’s, like when it’s really harsh and, yeah, and it’s like they’re sort of gasping as they’re coughing”.* (low-SES, 2 children 3 & 5y)

#### Child’s susceptibility

Several parents described perceived vulnerability or susceptibility when a child was particularly prone to coughs or suffered a lot compared to other children or siblings. Sometimes this was attributed to a particular event (previous illness), to something that ran in the family (genetic predisposition) or to a perceived underlying problem or weakness in the child’s immunity.

*“Weak chests, you know, um in my family, you know, most of my family have, you know, had asthma…. I never even thought that children could get pneumonia…. now I know that it’s possible, I’m obviously gonna be on the case every time she [] gets a snuffle or a cold or a slight cough, I wouldn’t hesitate to take her to the doctor…because she’s got a weakness now, hasn’t she, well like a vulnerability?” (low-SES, 2 children 6* &*11y)*

*“He had chicken pox at three months old, so he become quite unwell then… and then once he did suffer with all those colds and coughs I worried a bit more… his immune system was low and I was thinking, “Well why does he keep getting them?” One night he was really snuffly, he couldn’t really breathe, and I took him to the Walk in Centre then, and that’s who explained to me about his immune system.” (low-SES, 2 children 3* &*5y).*

#### Illness information

Parents sought information and advice about coughs from a range of sources including lay and professional people within their social network (family, friends, health visitors, nursery workers and pharmacists), written information and advice available on websites, books and leaflets. No single information source was used by all parents and they often referred to multiple sources before deciding what to do, if anything. When assessing the trustworthiness of information sources, parents felt that ‘professional’ sources were more credible. NHS branded websites were generally more trusted than other internet sources, as were people in their social network with some health training. However, those with extensive personal experience of children's illness were also trusted. Contradictory information or advice was likely to contribute to a decision to consult.

Social network:

*“I would go to either my sister (she has 5 daughters) or my gran and speak to them about it, see what they say and I’d sort of mention it to friends”.* (high-SES, 2 children 11 & 13y)

*“I would ask health professionals that are in the family. My aunt is a GP, so I would ask her”.* (mid-SES, 1 child 11m)

*“I just go to my mum, and she’s normally right”*. (low-SES, 1 child 5y)

Using the internet:

“*One site I used a lot when he was tiny was Baby Centre. I still go back, because they are quite – it’s a very small amount of information, and it’s very contained and it’s very matter of fact. It gives you sources of information to go to, but it’s not – it’s not like a forum”.* (high-SES, 1 child 11m)

*“Oh well, I would Google something and then I would look at something like Net Doctor or (Net Health)”.* (high-SES, 2 children 11 & 13y).

*“We will Google (…,) my daughter had a cough that she couldn’t shake off and we were starting to check symptoms and work on it before we went to the GP, but you want to be going to a reputable website, you don’t want somebody saying, “These symptoms mean your child can die.”* (mid-SES, 2 children 10 & 12y)

*“But sometimes it comes up with (…) worrying stuff (…) it can make it a bit scary sometimes just using Google, I reckon. And then when you look on the NHS it’s something like totally different. So I do try and tend to stick to the NHS one”.* (low-SES, 2 children 10m & 5y)

Other sources:

*“I’ve been to pharmacy about say a cough or something, rather than going to the doctor”* (high-SES 2 children 11 & 13)

*“My books are more accessible than my family and friends”.* (high-SES, 1 child 1y)

*“I’ve phone NHS Direct before, you know the non-emergency one? Like just to ask them for advice?*” (low-SES, 1 child 5y)

”*I usually go to the pharmacist near my work and ask him just ‘cos usually you can ask pharmacist there and then”*. (low-SES, 1 child 10m)

### Perceived triggers, benefits, and barriers to consulting for a cough

#### Consultation triggers

Fears and uncertainties were similar for parents from all demographic backgrounds and encouragement from others and their own uncertainty caused them to consult primary care.

#### Social pressures and sanctioning

Parents from all backgrounds mentioned feeling uncertain (about identifying and interpreting symptoms, child’s diagnosis), feeling that it was safer to consult if in doubt, and sanctioning or pressure from friends or family. Sometimes this sanctioning advice appeared to be a trigger for consultation when they might not otherwise have gone; sometimes it appeared to be a welcome confirmation that they were justified in consulting. Sanctioning was similar across parents from different backgrounds and with older and younger children.

*“ I think mostly, the only time I have been to the doctor is when I felt pressurised into going from my mum really, or other people who have said, “You must take her to the doctor, she sounds awful,” and then you go,”.* (mid-SES, 2 children 11 & 12y)

“…I’ve so far relied on the nursery telling me to go to the doctor. Because when he was getting really bad the nursery were like, “Well, you know, I’d get that seen by the doctor.” (low-SES, post grad, 1 child 1y)

#### Uncertainty and failure of home management

Parents talked about whether or not symptoms responded to home management and wanted guidance about potentially serious symptoms which needed to be seen by a doctor. They talked about both the severity of symptoms (how chesty the cough / how high the temperature) and the duration of symptoms (how long should a cough go on for before consulting the doctor) and their need for information and advice in relation to these could trigger consultation.

Over-the-counter medicines, home remedies and perceived failure of home management were all mentioned as key triggers to consultation. Sometimes it was specific ‘rules’, such as if the paediatric paracetamol (Calpol) doesn’t bring the temperature down, or if the child wasn’t better within a certain time frame.

*“I do remember recently administering cough medicine at home, and thinking to myself, “Is this the right cough medicine? Is it tickly or is it a chesty one?” (..)I think it would be quite useful to have a little bit more of an elaborate definition of what’s tickly and what’s chesty.” (mid-SES, 2 children 9* &*12y)*

*“After 24 hours of doing that, I suppose I might phone. But I might not. I’m not quite sure actually at what point. So I mean I would find it quite useful actually, what is the pattern of a chest infection.” (mid-SES, 2 children 10* &*13y)*

“You want to know how to soothe them. How I can just make it manageable.” (mid-SES, 1 child 11m)

#### Perceived benefits of the consultation

Once they had decided to consult primary care, most parents saw the benefit as receiving a medical evaluation of their child’s illness by a clinician. This was referred to as having a ‘proper check’ and was often described in terms of the physical examination, particularly the clinician listening to the child’s chest with a stethoscope. Parents believed that a clinician would be able to tell whether or not their child had a serious illness when the parents themselves were uncertain.

*“The safest bet is to talk to a proper doctor. Yeah it’s just peace of mind that, you know deep down in your heart that it’s probably only going to be a virus, but you just want for it to be double checked to make sure”. (low-SES, 3 children 6, 12* &*15y)*

“I was concerned that I wasn’t sure if it was a chest infection or not, so I wanted to get it checked out. […] I don’t really know what the symptoms of chest infection actually are.” (high-SES, 1 child 1 y)

Parents also wanted information to help them understand and support their management of the illness including signs of serious illness (when do I need to worry?), how to care for child (what might help, what to avoid?), what is normal, and how to prevent or reduce future episodes. This could be conceived as a benefit to them as they were reassured or gained knowledge.

“It was for me to establish whether that was a chesty cough or not, so that I would know for future.” (high-SES, 1 child 1y)

“The answer I want is at what point do I need to start worrying? – that’s the answer that you don’t ever seem to get”. (high-SES 1 child 11m)

Several parents were seeking or hoping for treatment or advice on treatment other than antibiotics, mainly to alleviate symptoms to reduce their child’s suffering and impacts on the child’s and family’s life. Others sought treatment to address a perceived chronic problem or underlying problem with the child’s immune system – something to boost the child’s immunity and reduce the frequency with which they had acute cough.

*“I’m looking for somebody to give me some advice on something I can do to alleviate the frequency that this child is having these illnesses. Any advice on how to increase her immune system, because this is just constant from November to January.” (high-SES, 2 children 4* &*6 y)*

“… some way of them not being sick, some magic cough mixture that would stop them reacting like that.” (high-SES, 2yr twins)

Some parents expected antibiotics from the consultation. A few were fairly confident they knew when their child had an RTI which needed antibiotics. This was usually based on past experience of their child having antibiotics for something similar, or remembering having antibiotics for similar illnesses themselves in childhood. A few parents believed that certain illnesses (tonsillitis, ear ache) needed antibiotics or that a specific child needed antibiotics when they got RTIs as they couldn’t recover themselves.

*“Because I’m unclear. (..) Now I don’t actually want to go to the doctor until I think it’s an infection. What is it about the cough? You know, there’s all these other coughs, is this a normal cough which presumably is viral and not an infection”. (mid-SES, 2 children 10* &*13y)*

#### Barriers to consulting

The few barriers raised by parents included feeling that they were wasting the doctors time as it was ‘only a cough’, the time and effort involved in getting to the surgery and bad previous experiences when a serious chest infection had been missed resulting in a loss of confidence in their doctor. However, they still may go to Accident & Emergency or the Walk in Centre if they were still worried by the illness.

*“Because I’d just been told it was cough all the time or a cold, you do feel like a right plonker, keep going back there with your child and saying, “Look, I know something’s wrong,” and they’re telling you, “No.” And you feel like you’re wasting their time. .. It does make you reluctant. And you think well I don’t want to waste their time. I don’t want them to be talking about me wasting their time, you know.” (low-SES, 2 children 2* &*11 y)*

*“And I just thought, I’m not going back to the doctors; no one is listening to me.” (low-SES, 2 children, 5m* &*5y)*

### Perceived parental efficacy, reassurance and experience

Experience was the key factor which parents reported increased their self-efficacy [21], that is their confidence that home care was likely to be successful and thus reduce their need to consult or re-consult the doctor. Less experienced parents described their difficulties in differentiating between serious and minor coughs and some admitted to finding the frequency with which young children suffered from cough a surprise – highlighting the role of un-realistic expectations and the need for more appropriate information.

Parents described how they were unable to obtain helpful information or advice to facilitate their decision to consult. In relation to cough, they expressed uncertainty about the way to distinguish between a “normal” cough which would be self-limiting and a cough which needed to be seen and treated by a doctor. They were then consulting because they were not sufficiently reassured by the information they had found and felt it safer to consult the doctor.

“I remember being in the doctor’s surgery three days running; it would be nice to know that quite early on, that actually your child is going to catch lots of coughs and colds. And I know you look back and think, yeah, I can kind of see that that would happen, but you don’t [realize]” (high-SES, 1 child 11m)

*“I don’t want a prescription every time I go, I just want reassurance that I’ve done the right thing in coming.” (high-SES, 2 children 7m* &*23m)*

*“The first year you look up a lot of things, then you sort of learn to treat the most usual things and the signs to alarm or not. (…) When you’re still learning about all these childhood non-serious illnesses it takes some learning”.* (mid-SES, 1 child 3y)

*“I think a lot of the time you just go by your instincts anyway, because you automatically know what your children are like and how bad they are. And once you’ve had the first one, it’s just experience then, I reckon”.* (low-SES, 2 children 10 & 12y)

Views were compared and across and between groups of different socio-economic status and with different ages of children. Although some differences emerged within groups, they did not differ substantially between the groups.

## Discussion

### Summary of main findings

Our study found that parents from all socio-economic backgrounds sought information from a wide range of sources about RTIs in children in order to identify which of their child’s symptoms they should worry about and trigger a visit to the doctor. The personal threat of a cough to a child as perceived by parents included a combination of the severity of the illness and the susceptibility of a particular child to developing a cough (as predicted by the health belief model). Information was also sought to support self-care and increase parental self-efficacy to care for their child at home. Parents sought specific advice about their child’s current circumstances, rather than general advice about when to consult. The role of friends and family as important sources of such information for parents across all groups was highlighted. Experience with other children increased perceived self-efficacy. Interestingly for our understanding of triggers to reconsultation (when parents return within the same illness episode), the consultation itself was seen to be beneficial regardless of treatment decisions, because it secured a medical evaluation, reassuring parents and providing them with knowledge. Relating our findings to the psychological models of health behaviour has helped to understand help seeking behaviour and reconsultations.

### Comparison with existing literature

Kai [2,6] (exploring acute childhood illness) and Francis [22] (for RTIs) included only parents from deprived populations in their studies, since they are known to be higher consulters in primary care, but our study shows that parents from all socio-demographic groups have similar concerns and needs. Kai’s study showed that disadvantaged inner city parents reported feeling under pressure when their child was ill and this was linked to their sense of personal control and the perceived threat of the illness [2,6]. Francis’s study [22] reported that parents commonly express a desire to have greater information about recognising serious illness in their children and his more recent study highlighted the difficulties that parents have in interpreting the importance of signs and symptoms of serious RTI which may cause delays in consulting [23]. Neill [24] also explored acute childhood illness within family life and similarly reported parents’ uncertainty about when to seek medical help; also confirmed in our study. Studies exploring internet use for health information have emphasised the need to guide parents regarding reliable resources online [25] and reported parental preferences for online clinical health information to be presented by clinical professionals, and online parenting advice to be presented by other parents [15].

Influences on parental help-seeking behaviour have been explored in common childhood problems in toddlers [26] and for child mental health concerns [27]. Consulting was linked to parental perception of illness severity [5], child’s susceptibility to the illness, perceived parental efficacy and ability to cope, their knowledge of the illness and the availability of services. Others have used health belief models to explain the psychological factors influencing help seeking consulting behaviour [10,11], or have modified it to reflect a health promotion stance for young families [28]. This latter approach reoriented the health belief model and based it on positive health definitions associated with health promotion.

Parental focus on the specific features of this illness episode (susceptibility to this type of illness, severity of this episode) could explain why the health belief model has been less successful in explaining take up of routine care [29]. Our study illustrates that a combination of: the perception of the severity of this illness; together with social and factual information sources; and parental efficacy, informs parents’ decisions about whether to consult the doctor.

### Strengths and weaknesses

Ours is the first study to explore these issues in the context of acute childhood RTI with a range of parents from different socioeconomic backgrounds, enabling a complete picture of views. Although some differences emerged within groups, they did not differ substantially between the groups. Findings from the focus groups were then explored further in the interviews particularly around illness susceptibility and severity and the costs and benefits of consulting. Views were almost exclusively those of mothers since only two fathers attended for interview and none participated in the focus groups, so this study was unable to make meaningful comparisons between the experiences of mothers and fathers. The data analysis was robust as coding was independent, attention was given to contradictory views and themes arising were discussed within the research group.

### Implications for future research or clinical practice

The commonality of parental information needs across socio-economic groups, along with the wide variety of information sources currently used, suggests that parents would value consistent advice from a trusted source which addresses their common concerns and supports home care and decision making about seeking help. These factors should be taken into consideration by researchers, clinicians and health care providers*.* Parents want to understand their child’s illness better, and be reassured that they are not seriously ill (either currently or having an underlying illness). The impact of such information on parental self-efficacy and subsequent use of health care services should be evaluated.

## Conclusions

Parental perceptions influence their behaviour in respect to whether they view their child’s cough to be serious enough to consult their GP and these perceptions are similar across all socio-economic groups. Parents’ experience, confidence and efficacy also influence the likelihood of consulting primary care for their child’s cough. Parents value the consultation in itself for reassurance and information provided. Clinicians across all sectors of the healthcare system should provide consistent information that promotes parental self-efficacy in the care of their unwell child.

### Additional information

Ethical approval from Southmead Local Research Ethics Committee 10/H0102/55.

## Competing interests

The authors declare that they have no competing interests.

## Authors’ contributions

JI, JH and AH were responsible for developing the research questions and study design; JI, CC, JH for study management and with PLu writing the manuscript; and AH, MT, NR for commenting upon the final manuscript; JI accepted the final version. All authors read and approved the final manuscript.

## Pre-publication history

The pre-publication history for this paper can be accessed here:

http://www.biomedcentral.com/1471-2296/14/106/prepub
